# Temperature- and Nutrients-Induced Phenotypic Changes of Antarctic Green Snow Bacteria Probed by High-Throughput FTIR Spectroscopy

**DOI:** 10.3390/biology11060890

**Published:** 2022-06-09

**Authors:** Margarita Smirnova, Valeria Tafintseva, Achim Kohler, Uladzislau Miamin, Volha Shapaval

**Affiliations:** 1 Faculty of Science and Technology, Norwegian University of Life Sciences, 1432 Aas, Norway; valeria.tafintseva@nmbu.no (V.T.); achim.kohler@nmbu.no (A.K.); volha.shapaval@nmbu.no (V.S.); 2 Faculty of Biology, Belarussian State University, 220030 Minsk, Belarus; vladmiamin@mail.ru

**Keywords:** green snow bacteria, cellular chemical profile, FTIR spectroscopy, multivariate data analysis

## Abstract

**Simple Summary:**

Green snow microorganisms play an important role in biogeochemical cycle and carbon sink processes and they can be a source of biotechnologically interesting cell factories. A wide temperature tolerance is a unique property of bacteria isolated from cold environments, which has received great attention in the last years. The present paper examines the growth and chemical profile flexibility for green snow bacteria exposed to different temperature and nutrient fluctuations. By applying high-throughput chemical phenotyping with FTIR spectroscopy we discovered chemical changes possessed by green snow bacteria when grown at high/low temperature and rich/minimal media.

**Abstract:**

Temperature fluctuations and nutrient composition are the main parameters influencing green snow microbiome. In this study we investigated the influence of temperature and nutrient conditions on the growth and cellular chemical profile of bacteria isolated from green snow. Chemical profiling of the green snow bacteria was done by high-throughput FTIR spectroscopy combined with multivariate data analysis. We showed that temperature and nutrients fluctuations strongly affect growth ability and chemical profile of the green snow bacteria. The size of colonies for green snow bacteria grown at higher (25 °C) and lower (4 °C and 10 °C) than optimal temperature (18 °C) was smaller. All isolates grew on rich medium, and only 19 isolates were able to grow on synthetic minimal media. Lipid and mixed spectral regions showed to be phylogeny related. FTIR fingerprinting indicates that lipids are often affected by the temperature fluctuations. Growth on different media resulted in the change of the whole chemical profile, where lipids showed to be more affected than proteins and polysaccharides. Correlation analysis showed that nutrient composition is clearly strongly influencing chemical changes in the cells, followed by temperature.

## 1. Introduction

Climate change and global warming lead to the accelerated melting of glaciers, coastal snow fields, and overall decrease in the snow cover in Antarctica. This creates favorable conditions for blooming of snow algae, resulting in the appearance of green snow areas. The blooming of snow algae in Antarctica was recorded for the first time during expeditions in the 1950s [1]. Due to that, warming in the Antarctic Peninsula has already exceeded 1.5 °C [2,3] and an increased spreading of green snow areas throughout the continent has been recorded. During the spring-summer periods, green snow areas can occupy hundreds of square meters [4]. Thus, it is an important ecosystem with a role in the global biogeochemical cycle and terrestrial carbon sink [5].

Green snow is characterized by the presence of a complex microbiome, where algae are the main colonizers and bacteria, archaea, and fungi are co-colonizing microorganisms [6,7,8]. Green snow is a highly mobile ecosystem with continuously changing physicochemical parameters, where temperature and nutrient composition are the main parameters influencing green snow microbiome. While green snow algae have been studied extensively, bacteria co-colonizing green snow have been studied to a very limited extent. In our recent study [9] we performed identification and complex phenotypic characterization of 45 fast-growing bacterial isolates from green snow collected during the 7th Belarussian Antarctic expedition (2014–2015). 16S rRNA sequencing of the isolated green snow bacteria identified their belonging to the phyla *Actinobacteria* and *Proteobacteria*, and genera *Arthrobacter*, *Cryobacterium*, *Leifsonia*, *Salinibacterium*, *Paeniglutamicibacter*, *Rhodococcus*, *Polaromonas*, *Pseudomonas*, and *Psychrobacter*. Interestingly, despite originating from the cold environment, all green snow bacteria were characterized by a wide range of temperature tolerance from 4 °C to 25 °C, and the optimum growth temperature of 18 °C.

A wide temperature tolerance is a unique property of bacteria isolated from cold environments that has received great attention in the last years [10,11]. For example, low temperature tolerant bacteria, called psychrophilic, could be a source of cold-active enzymes, polyunsaturated fatty acids, exopolysaccharides, cold-shock, cold-acclimation, and antifreeze proteins with the applications in agriculture, medicine, and biotechnology [12,13,14]. In addition, some psychrophilic bacteria from phyla *Proteobacteria* and *Bacteroides* can degrade hydrocarbons and are applied in the biodegradation of oils and petroleum and bioremediation as a treatment of contaminated wastewater [14,15,16]. Adaptation of bacteria to different temperatures is a complex cellular process involving several metabolic pathways, often resulting in changes of chemical phenotype. For example, it has been shown that bacteria isolated from ice and melted snow possess changes in the membrane lipid composition, enzyme secretion, and pigments accumulation when grown under different temperatures [17,18,19,20,21]. Nutrient composition and availability may influence the temperature adaptation mechanisms in bacteria. Thus, it has been reported that mineral composition of growth medium affected the synthesis of cold-adaptive proteases in *Pseudomonas arctica* isolated from the soil in King George Island, Antarctica [22]. Due to the rising temperature fluctuations in Antarctica, understanding temperature and nutrient-induced changes of chemical phenotype of green snow bacteria could provide forecasting information, predicting their further contribution to the biogeochemical cycle. In addition, these chemical changes might be interesting from a biotechnological point of view as they could be represented by the accumulation and production of highly valuable chemical components.

The main aim of this study was to examine, in detail, the influence of different temperatures and nutrients on the growth ability and cellular chemical profile of the Antarctic fast-growing green snow bacteria. Bacteria were grown on rich complex medium—brain heart infusion (BHI) and minimal media with the different carbon sources and mineral composition. Growth on the selected media was done at 4, 10, 18, and 25 °C. For the profiling of the cellular chemical profile, Fourier transform infrared (FTIR) spectroscopy was utilized. FTIR spectroscopy is a rapid, non-destructive next-generation phenotyping technique widely used in medicine [23,24,25,26,27,28], food industry [29,30], and biotechnology [31,32,33,34] for discrimination, identification, and characterization of different microorganisms [9,33,35,36,37,38,39,40,41,42,43,44,45,46,47,48,49,50]. FTIR spectroscopy can be applied in a high-throughput set-up and there are automated sample preparation systems for FTIR available [51,52,53]. Previously, we successfully applied this technique for the biochemical fingerprinting of the same bacterial strains cultivated on broth and agar media and identified a possible accumulation of intracellular polymer polyhydroxyalkanoates (PHA) and lipids for some isolates [9]. In this study we combined FTIR spectroscopy with the multivariate data analysis for detecting temperature and nutrient-induced changes of the chemical phenotype of the Antarctic green snow bacteria.

## 2. Materials and Methods

### 2.1. Antarctic Green Snow Bacterial Isolates

Forty-five fast-growing bacteria were isolated from the two green snow samples collected during the 7th Belarussian Antarctic Expedition (2014–2015). Identification of the isolated bacteria was performed by 16S rRNA gene sequencing, and characterization of enzymatic activity, optimum growth temperature and biochemical phenotyping by FTIR spectroscopy was reported previously [9]. Bacterial isolates were deposited at the Belarussian Collection of Non-pathogenic Microorganisms (Institute of Microbiology of the National Academy of Sciences of Belarus) (Table 1).

### 2.2. Cultivation of Bacteria

Cultivation of bacteria was performed on five agar media: complex medium brain heart infusion (BHI) (Sigma-Aldrich, India), two types of minimal media with different nitrogen and phosphorus content and two different carbon sources, glucose and glycerol. For preparing minimal media, two types of salt solutions, M9 [54] and a modification of M9 salts solution—X9, were used. M9 stock solution was prepared as follows (g/L): Na_2_HPO_4_—24, KH_2_PO_4_—15; NaCl—2.5; NH_4_Cl—5. MGU minimal medium was prepared by mixing of 300 mL of 20% agar, 100 mL of M9 stock solution, 4 mL of 20% glucose, 4 mL of 0.1 M (CaCl_2_) and 4 mL of 0.1 M (MgSO_4_). X9 stock solution was prepared as follows (g/L): NH_4_Cl—20; NH_4_NO_3_—4; Na_2_SO_4_ × 10H_2_O—8; K_2_HPO_4_—12; KH_2_PO_4_—4; MgSO_4_ × 7H_2_O—0.4. XGU minimal medium was prepared by mixing of 300 mL of 20% agar, 100 mL of X9 salt stock solution and 4 mL of 20% glucose. All media components were autoclaved at 115 °C for 15 min. Two analogous minimal media, MGY and XGY, with glycerol as carbon source were prepared by added 4 mL of 20% glycerol instead of glucose (Table 2).

Cultivations were performed at four temperatures: 4 °C, 10 °C, 18 °C, and 25 °C by streaking cultures on all five agar-based media. The ability to grow on different media and at different temperatures was determined by daily visual evaluation of the growth at four areas of the streak plates: (1) area of inoculation and first streaks, (2) area of second streaks, (3) area of third streaks, and (4) area of fourth streaks. The evaluation of the growth was performed according to the score-based scale were score “0” indicates the absence of growth on all areas of the streak plates, score “1–2” indicates a growth only at the area of inoculation and first streaks but without single colonies, score “3–4” indicates a growth at the area of inoculation and first streaks and area of second streaks with single colonies below 0.5 mm in diameter, score “5–6” was for the growth at all areas of the streak plates with single colonies 0.5–1.0 mm in diameter, score “7–8” when the diameter of the colonies was 1.5–2.0 mm, and score “9–10” for colonies with a diameter higher than 2.5 mm. The cultivation time was for 3–12 days depending on the cultivation conditions and strains (Table S1). Agar cultivation for FTIR phenotyping of green snow bacteria was chosen due to the better growth than in broth media and higher differences in cellular biochemical profile for some isolates, as reported previously [9]. All agar cultivations were done in three biological replicates—independent agar cultivations performed on different days.

### 2.3. FTIR Spectroscopy Analysis

Sample preparation for FTIR spectroscopy measurements was done as previously described [9]: (1) bacterial biomass was collected from agar plates after cultivation and washed three times with 50–100 µL of distilled water by applying centrifugation at 25,200× *g*, 4 °C for 5 min; (2) at the last washing step, 50–100 µL of distilled water was added for resuspending the cell pellet; (3) 8–10 µL of the obtained cell suspension was transferred onto IR-light-transparent silicon 384-well microplate (Bruker Optics GmbH, Ettlingen, Germany) in three technical replicates; (4) samples were dried at room temperature for 1 h.

FTIR spectroscopy analysis was performed using the High-throughput Screening eXTension (HTS-XT) unit coupled to the Vertex 70 FTIR spectrometer (both Bruker Optics GmbH, Ettlingen, Germany) allowing to perform High-throughput Screening (HTS) transmission mode measurements. The spectra were recorded in the region between 4000 and 500 cm^−1^ with a spectral resolution of 6 cm^−1^ and an aperture of 5.0 mm.

### 2.4. Data Analysis

Prior to data analysis, the spectral data were quality checked. A quality test has been applied to pre-select good quality spectra for further analysis. This was done using the method published by Tafintseva et al. [55,56,57]. The quality checked spectra were preprocessed and analyzed by multi-block PCA [58,59,60,61] as described further. For data modelling, spectra were split into the following regions: lipid region at 3050–2800 cm^−1^ and 1800–1700 cm^−1^, protein region at 1700–1500 cm^−1^, mixed region at 1500–1200 cm^−1^, and polysaccharide region at 1200–700 cm^−1^. Each of the spectral regions were preprocessed separately as follows: (1) second derivative spectra were obtained by the Savitzky–Golay algorithm [62,63] with window sizes of 13, 17 and 21 for lipid, mixed, and protein regions, respectively, and with window size 13 for the polysaccharide region, using a polynomial of second order; (2) spectra were normalized by Extended Multiplicative Signal Correction (EMSC) with linear and quadratic terms [64,65]; (3) average of all replicates was calculated providing one spectrum per strain and each temperature condition. Further, multi-block PCA was applied separately on each spectral region fusing data of all five media. Thus, the data of each medium represented a separate block in multi-block PCA. Three multi-block PCA models were built, with one for each spectral region. Multi-block analysis requires sample-to-sample correspondence across different blocks of data. Averaging of spectral replicates that cannot be coupled to each other from different blocks is a prerequisite necessary for meaningful multi-block analysis. In our case, samples from different media should correspond to each other. Replicates were, therefore, averaged and samples which were not available in one of the five blocks (for example due to poor growth or poor spectral quality) were removed completely from the multi-block PCA analysis. In total, 67 samples were used in multi-block PCA after averaging and data fusion.

Correlation analysis was done to detect the effect of nutrients and temperature on different strains. To do that, all the data representing different growth conditions were analyzed together (put together as rows), as opposed to multi-block setting where data is put next to each other as separate blocks (put together as columns). The spectra were preprocessed as follows: the spectral regions 3050–2800 and 1800–900 cm^−1^ were selected, and spectra were normalized by EMSC [64,65]. PCA analysis was done on the preprocessed spectra of each genus separately and correlation loading plots were obtained to find correlations between design parameters such as temperature and media, and spectral variables. Only the subset of spectral variables (peaks) was plotted on the correlation loading plots. To find peaks, the minima were obtained by using the second derivative of the Savitzky–Golay algorithm [62] with 13, 17, 21, and 13 windows sizes for lipid, mixed, protein, and polysaccharide regions, respectively, and second order polynomial. For some genera we had only spectra from BHI medium since there was no growth on the minimal media observed. For the correlation analysis the following IR peaks were selected: for the lipid region—2962 cm^−1^, 2927 cm^−1^, 2925 cm^−1^, and 2854 cm^−1^ peaks representing –C–H (CH_3_)/–C–H (CH_2_)/CH_2_/CH_3_ stretching, and 1745 cm^−1^, 1743 cm^−1^, and 1741 cm^−1^ representing ester bond stretching, for mixed—1456 cm^−1^, 1454 cm^−1^, 1394 cm^−1^, 1380 cm^−1^, 1338 cm^−1^, 1336 cm^−1^, 1313 cm^−1^, 1311 cm^−1^, and 1305 cm^−1^ peaks representing CH_2_/CH_3_ bending modes or deformation of this functional groups modes, 1404 cm^−1^, 1402 cm^−1^, and 1400 cm^−1^ representing C=O symmetric stretching vibrations of COO^−^ functional groups and 1243 cm^−1^, 1242 cm^−1^, and 1240 cm^−1^ representing –P=O stretching vibrations of phosphodiesters; and, for proteins, 1654 cm^−1^, 1548 cm^−1^, and 1546 cm^−1^ peaks characterizing the Amide I and Amide II bands of proteins [66]. Peaks from the polysaccharide region were not included in the correlation analysis as we did not observe any nutrient- and temperature-induced changes when using this region.

## 3. Results

### 3.1. Growth Ability under Different Nutrient and Temperature Conditions

The ability of the green snow bacteria to grow on different media and temperatures was analyzed by the daily growth evaluation and it was expressed by the score-based scale (Table 3). All bacterial isolates showed a good growth on rich complex BHI agar at 18 °C with colonies in the diameter from 1.5 to 2.0 mm to higher than 2.5 mm depending on the isolate, except *Polaromonas* sp. BIM B-1676, which had relatively small size colonies of about 0.5–1.0 mm in a diameter. Cultivation of isolates on BHI agar at 10 °C and 4 °C provided slightly smaller colonies and, in some cases, a considerable growth reduction was observed, as for example for *Salinibacterium* isolates (Table 3). Similar results were obtained for bacteria grown on BHI at 25 °C, where there was a noticeable decrease in the colony size for all green snow bacteria, except for *Pseudomonas* isolates, and some of *Rhodococcus* and *Arthrobacter* isolates. Interestingly, *Pseudomonas* isolates, when grown on BHI agar, were characterized by the equally good growth ability at all tested temperatures.

Cultivating the green snow bacteria on synthetic minimal agar media showed that all isolates of genera *Arthrobacter*, *Pseudomonas*, *Rhodococcus* (except *Rhodococcus yunnanensis* BIM B-1621), and isolate *Leifsonia antarctica* BIM B-1671 had a good growth, while all isolates of *Cryobacterium*, *Paeniglutamicibacter*, *Polaromonas*, *Psychrobacter*, *Salinibacterium*, *Leifsonia* (except *Leifsonia antarctica* BIM B-1671), and isolate *Rhodococcus yunnanensis* BIM B-1621 had little to no growth (Table 3). Generally, the size of colonies for bacteria grown on synthetic minimal agar was smaller than when grown on rich complex BHI agar, except for *Pseudomonas* isolates, which showed equally good growth ability on all media and temperatures. Similarly, as on BHI agar, an increase in the temperature up to 25 °C resulted in smaller colonies than at lower temperatures (Table 3). Isolates of *Arthrobacter* could be characterized by a quite diverse isolate-specific growth ability, where isolates *Arthrobacter cryoconiti* BIM B-1627 and *Arthrobacter* sp. BIM B-1666 were not able to grow on minimal media at 25 °C and *Arthrobacter oryzae* BIM B-1663 was not able to grow on minimal media at 4 °C. All isolates were able to utilize glucose and glycerol, except *Paeniglutamicibacter antarcticus* and *Salinibacterium* sp., which, according to the literature, are not able to utilize these carbon sources [67,68,69]. Interestingly, isolate *Leifsonia antarctica* BIM B-1671 grew well on the minimal media at lower temperatures but not at 25 °C, while other *Leifsonia* isolates were not able to grow at minimal media.

In addition to the growth ability, we evaluated the cultivation time, which differed depending on the type of the medium and temperature. Cultivation on BHI agar showed faster growth than cultivation on the minimal media (Table S1). The fastest growth was detected for almost all bacteria cultivated on BHI agar at the optimum growth temperature of 18 °C (Table S1), while cultivation on the minimal media had the longest growth time, especially at low temperature (Table S1).

### 3.2. Chemical Profile Changes under Different Temperatures and Media

FTIR spectroscopy chemical profiling was performed for all isolates cultivated on BHI agar and cultivation on the minimal media was done only for 19 isolates from the genera *Arthrobacter* (9 isolates), *Pseudomonas* (6 isolates), *Rhodococcus* (3 isolates), and *Leifsonia antarctica* BIM B-1671 since other 26 isolates from genera *Crybacterium* (6), *Salinibacterium* (4), *Psychrobacter* (3), *Paeniglutamicibacter antarcticus* BIM B-1657, *Polaromonas* sp. BIM B-1676 and *Rhodococcus yunnanensis* BIM B-1621 (1) were not able to grow on these media.

In order to get a general overview over the chemical differences based on phylogeny, we performed multi-block principal component analysis (also called consensus PCA or CPCA [70]) using different media as blocks. CPCA analysis was done separately for different IR spectral regions: lipid, protein, mixed, and polysaccharide. The CPCA models were built using the averaged spectra of the samples’ replicates, both biological and technical. Thus, one spectrum is obtained for each strain cultivated under one growth condition. This was due to the nature of the multi-block analysis where each sample in one block, representing one medium, should correspond to one sample in another block. Since many strains did not grow on all five media, many spectra were removed, and only those which were obtained for all media were used for the analysis.

**Figure 1:**
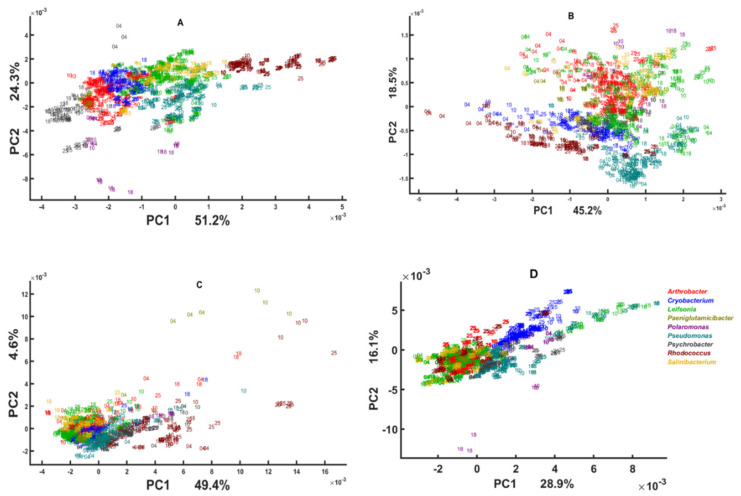
CPCA scatter plot of IR spectra of the green snow bacteria grown on BHI medium. The scores are calculated by using the BHI block of the multi-block PCA model built on the (**A**) lipid region (3050–2800 cm^−1^ and 1800–1700 cm^−1^); (**B**) mixed region (1500–1200 cm^−1^); (**C**) protein region (1700–1500 cm^−1^); and (**D**) polysaccharide region (1200–700 cm^−1^). Numbers correspond to the temperatures used in the study.

**Figure 2:**
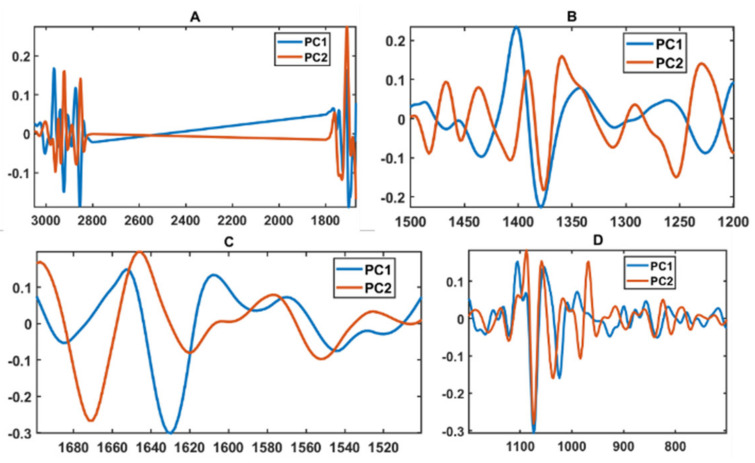
CPCA loading plots of IR spectra of the green snow bacteria grown on BHI medium. (**A**) Lipid region (3050–2800 cm^−1^ and 1800–1700 cm^−1^); (**B**) mixed region (1500–1200 cm^−1^); (**C**) protein region (1700–1500 cm^−1^); (**D**) polysaccharide region (1200–700 cm^−1^).

The BHI medium block scores of three multi-block models are presented in Figure 1 and loadings on Figure 2. To obtain these plots with isolates of all genera present, all samples with their replicates were projected onto the multi-block block scores corresponding to BHI medium. As it can be seen, the multi-block scores obtained from the lipid spectral region (Figure 1A) provide the best phylogeny-based separation on the genus level, where bacterial isolates of the genera *Rhodococcus*, *Psychrobacter*, *Pseudomonas*, and *Polaromonas* are clustered out from the main cloud formed by *Cryobacterium*, *Leifsonia*, *Arthrobacter*, *Paeniglutamicibacter*, and *Salinibacterium* isolates. In the main cloud we see that *Arthrobacter* and *Cryobacterium* isolates organized into two distinct clusters close to each other and *Cryobacterium* isolates clustered close to the *Leifsonia*, *Paeniglutamicibacter*, and *Salinibacterium* isolates, which did not show any clear clustering and were completely overlapping (Figure 1A). It should be noted that, while *Rhodococcus* and *Polaromonas* isolates are distinctly clustered out of the main cloud, isolates of *Psychrobacter* were closely positioned to *Arthrobacter* and *Pseudomonas* to *Leifsonia* isolates. BHI block scores of the mixed region shows a different clustering where only *Rhodococcus* and *Pseudomonas* are distinctly clustered out of the main cloud and *Cryobacterium* and *Arthrobacter* isolates are clearly separated, while overlapping with *Leifsonia*, *Paeniglutamicibacter*, *Polaromonas*, *Psychrobacter,* and *Salinibacterium* isolates (Figure 1B). CPCA loading plots for the lipid region (PC1/PC2) identify lipid-associated peaks responsible for the obtained clustering: (1) –C–H stretching vibrations of –CH_3_ and >CH_3_ at 2955 cm^−1^, 2869 cm^−1^, and 2937 cm^−1^, respectively [71]; (2) –C–H stretching vibrations of –CH_2_ at 2925 cm^−1^ and 2854 cm^−1^; (3) C=O stretching at 1743 cm^−1^ and 1739 cm^−1^ (Figure 2A). Also, lipid-associated bands such as –C–H stretching bands (1408 cm^−1^ and 1377 cm^−1^) identified to be significant on the loading plots for the mixed region. In addition, PO^−2^ asymmetric stretching of phosphodiesters in phospholipids at ~1230 cm^−1^ (PC1) and ~1250 cm^−1^ (PC2) is shown to be significant in this region (Figure 2B).

Multi-block analysis of the protein region does not show any discriminative clustering in the BHI block scores and only some species of *Rhodococcus*, *Paeniglutamicibacter*, *Arthrobacter*, and *Cryobacterium* grown on certain temperatures were clustered out (Figure 1C), which could be due to some differences in proteins structure and/or profile, as the loading plots identify several peaks in the Amide I (1620–1700 cm^−1^) and Amide II (~1550 cm^−1^) region as significant ones (Figure 2C). Similarly, as for the protein region, the polysaccharide region did not provide a clear phylogeny-based differentiation of the green snow bacteria, and only bacteria from genera *Cryobacterium*, *Pseudomonas*, and *Polaromonas* and some *Leifsonia* isolates were clustered out of the main cloud (Figure 1D). Loading plots of the polysaccharide region identified only P=O stretching band at 1078 cm^−1^ related to phospholipid and phosphodiester compounds [71] and some peaks of C–O–C and C–O ring vibrations in various polysaccharides (1120 cm^−1^, 1037 cm^−1^ (PC2) and 1024 cm^−1^ (PC1), and 980 cm^−1^) (Figure 2D).

Interestingly, the CPCA analysis shows that *Rhodococcus* and *Cryobacterium* isolates have very different lipid profile (Figure 1A), while the polysaccharide and protein profile of these isolates looks more similar (Figure 1C,D).

All spectral regions show significant temperature-based differences for isolates of different genera. Thus, for the lipid region, *Psychrobacter*, *Pseudomonas* and *Rhodococcus* isolates cultivated at 25 °C are separated from the ones grown at the lower temperatures (Figure 1A). For the mixed region, all the isolates cultivated at lower temperatures 4 °C and 10 °C clustered separately from the ones cultivated at higher temperatures (Figure 1B). For the protein region, temperature-induced differences can be observed for *Paeniglutamicibacter* isolates cultivated on 4 °C and 10 °C, and some *Rhodococcus* isolates cultivated on 4 °C, 10 °C, and 25 °C (Figure 1C). Cultivation at higher temperatures such as 25 °C and 18 °C for some isolates from genera *Cryobacterium*, *Rhodococcus*, and *Polaromonas* resulted in chemical differences observed at polysaccharide spectral region (Figure 1D).

**Figure 3:**
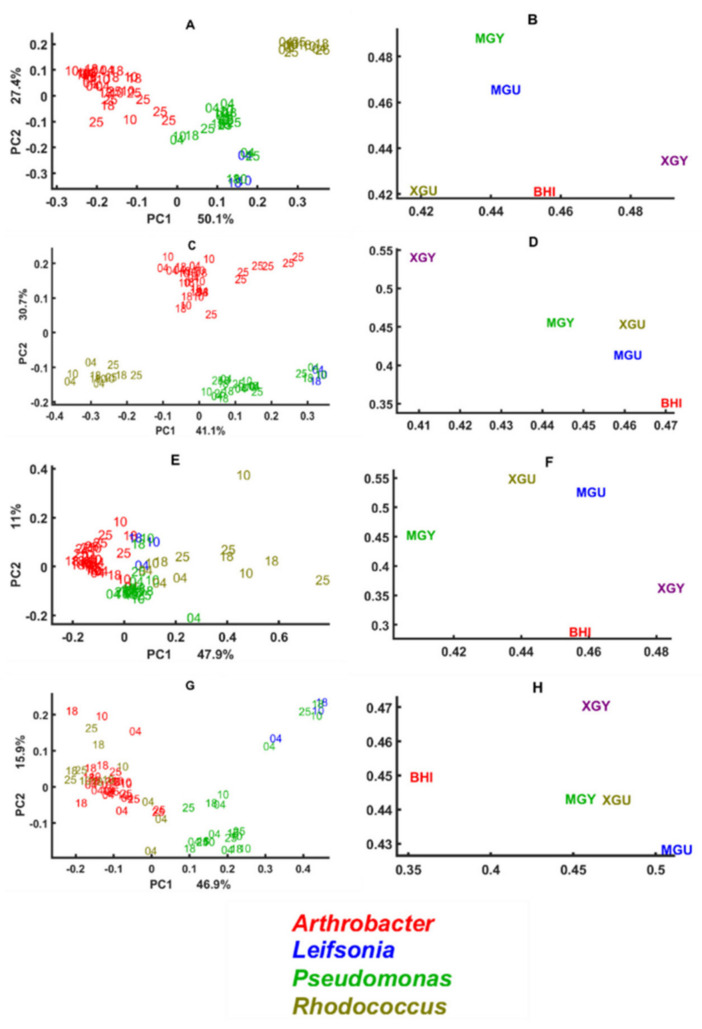
Global scores and super weights for multi-block models using lipid (**A**,**B**), mixed (**C**,**D**), protein (**E**,**F**), and polysaccharide (**G**,**H**) spectral regions. Different media represent different blocks in a multi-block model. Numbers 04, 10, 18, and 25 correspond to the cultivation temperatures. Colors on the plots A, C, E, and G correspond to the genera: red—*Arthrobacter*, blue—*Leifsonia*, green—*Pseudomonas*, olive—*Rhodococcus*. Other genera were not represented since they did not grow under these conditions.

**Figure 4:**
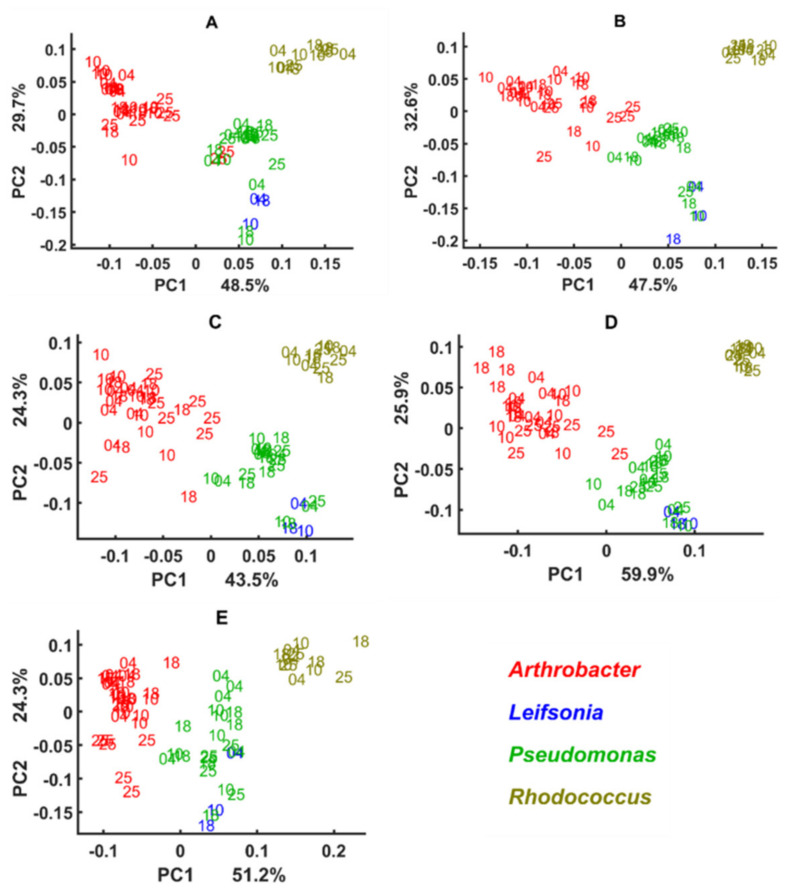
Block scores of the multi-block models built using lipid region of bacteria cultivated on different media and temperatures. Different media represent different blocks in a multi-block model: (**A**) MGU, (**B**) MGY, (**C**) XGU, (**D**) XGY, (**E**) BHI. Numbers 04, 10, 18, and 25 correspond to the cultivation temperatures. Colors on the plots correspond to the genera: red—*Arthrobacter*, blue—*Leifsonia*, green—*Pseudomonas*, olive—*Rhodococcus*.

**Figure 5:**
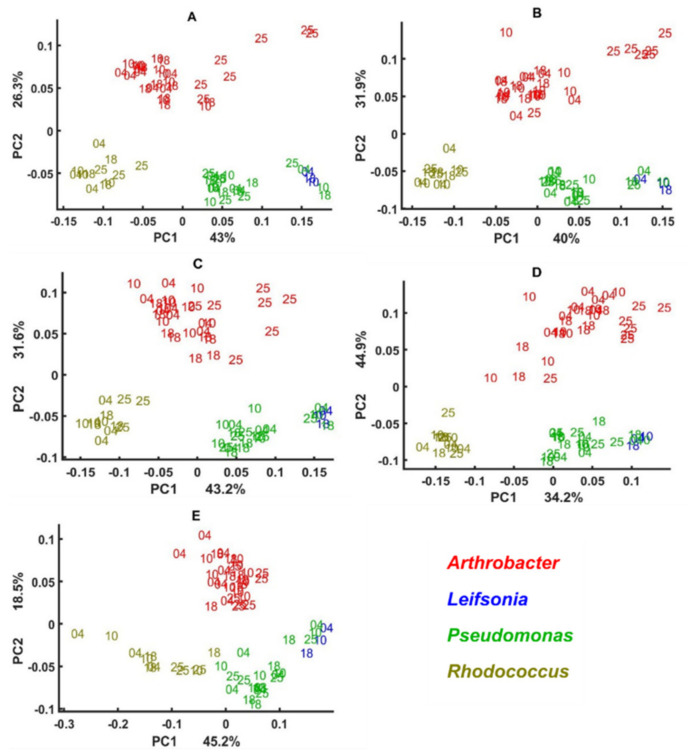
Block scores of the multi-block models built using a mixed region of bacteria cultivated on different media and temperatures. Different media represent different blocks in a multi-block model: (**A**) MGU, (**B**) MGY, (**C**) XGU, (**D**) XGY, (**E**) BHI. Numbers 04, 10, 18, and 25 correspond to the cultivation temperatures. Colors on the plots correspond to the genera: red—*Arthrobacter*, blue—*Leifsonia*, green—*Pseudomonas*, olive—*Rhodococcus*.

**Figure 6:**
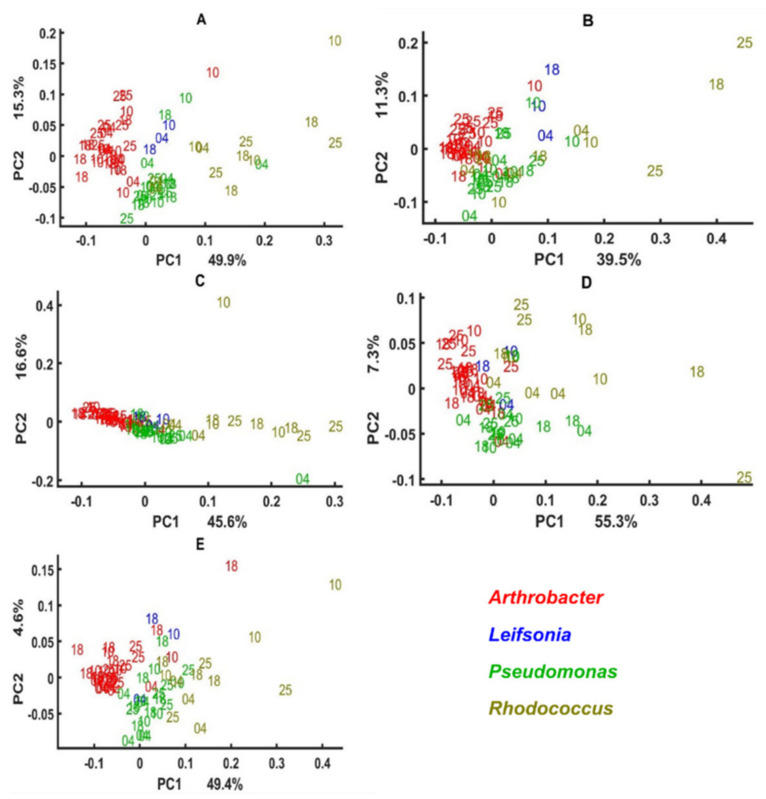
Block scores of the multi-block models built using the protein region of bacteria cultivated on different media and temperatures. Different media represent different blocks in a multi-block model: (**A**) MGU, (**B**) MGY, (**C**) XGU, (**D**) XGY, (**E**) BHI. Numbers 04, 10, 18, and 25 correspond to the cultivation temperatures. Colors on the plots correspond to the genera: red—*Arthrobacter*, blue—*Leifsonia*, green—*Pseudomonas*, olive—*Rhodococcus*.

**Figure 7:**
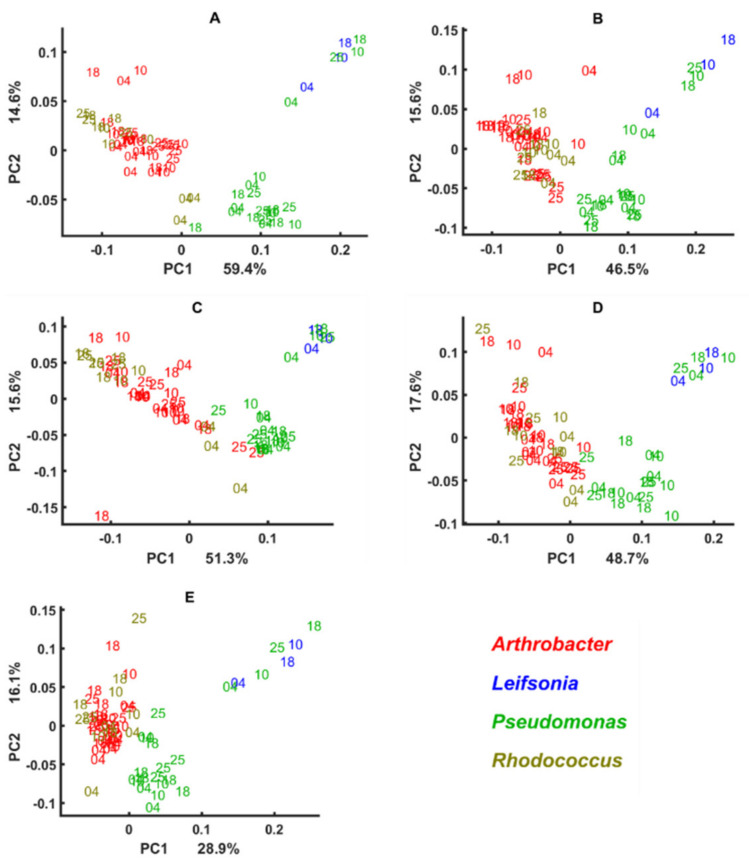
Block scores of the multi-block models built using polysaccharide region of bacteria cultivated on different media and temperatures. Different media represent different blocks in a multi-block model: (**A**) MGU, (**B**) MGY, (**C**) XGU, (**D**) XGY, (**E**) BHI. Numbers 04, 10, 18, and 25 correspond to the cultivation temperatures. Colors on the plots correspond to the genera: red—*Arthrobacter*, blue—*Leifsonia*, green—*Pseudomonas*, olive—*Rhodococcus*.

**Figure 8:**
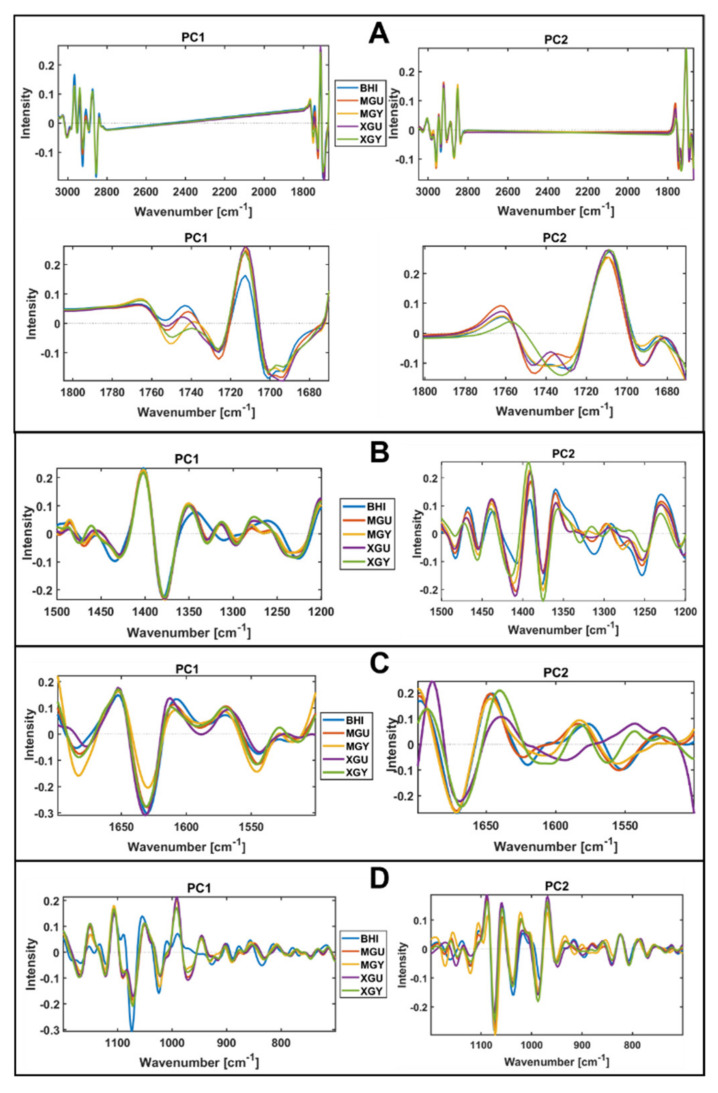
Block loading plots for PC1 and PC2 of IR spectra of the green snow bacteria grown on different media. (**A**) Lipid region (3050–2800 cm^−1^ and 1800–1700 cm^−1^); (**B**) mixed region (1500–1200 cm^−1^); (**C**) protein region (1700–1500 cm^−1^); (**D**) polysaccharide region (1200–700 cm^−1^).

Global scores and supper weights of three multi-block models of lipid, mixed, protein, and polysaccharide regions of the spectra of *Arthrobacter*, *Leifsonia*, *Pseudomonas* and *Rhodococcus* isolates cultivated on all agar media are presented in Figure 3, while the models’ block scores are presented in Figure 4, Figure 5, Figure 6 and Figure 7 and loading plots for the models’ block scores are presented in Figure 8. The other genera are not represented since they did not grow under these conditions and therefore could not be analyzed in a multi-block setting. Global scores represent the samples’ pattern in a global space of the multi-block model, while the weights plot suggests what blocks contributed most to this pattern. The global scores and weights are shown for the first principal components (PC1 ad PC2). The media far along the x-axis are the highest, contributing to the pattern in the global scores along the first component, PC1. The media far along the y-axis are the highest, contributing to the pattern in the global scores along the second component, PC2. Generally, the global score plots show that lipid and mixed spectral regions provide the best phylogenetic separation of the isolates on the genus level (Figure 3A,C). Somewhat worse separation was observed for the protein region, and use of the polysaccharide region showed the least discrimination on the genus level, where only *Pseudomonas* isolates clustered out (Figure 3E,G). One isolate of *Pseudomonas* overlaps with the *Leifsonia* isolate (Figure 3A,C,G) and shows a considerably different lipid, mixed, and polysaccharide profile from other *Pseudomonas* isolates. In addition, for this *Pseudomonas* isolate, significant temperature-induced changes were observed at the lipid region, where the lipid profile of the cells cultivated at 4 °C and 25 °C is different from the ones obtained after cultivation at 10 °C and 18 °C (Figure 3A,C). A slightly less pronounced, but similar results can be observed for this strain in a mixed region: the cells cultivated at 4 °C and 10 °C differ from the ones obtained at 25 °C and 18 °C (Figure 3B). Further, we can see that *Arthrobacter* isolates possess the highest variations in lipid profile when cultivated at 25 °C (Figure 3A,C). *Rhodococcus* isolates and overlapping isolates of *Pseudomonas* and *Leifsonia*, when grown at 4 °C, exhibit some distortions in polysaccharides (Figure 3D).

The difference in the score patterns was mostly induced by the media XGY and MGY for the lipid region (Figure 3B), BHI and XGY for the mixed region (Figure 3D), XGY and XGU for the protein region (Figure 3F), and BHI and minimal media for the polysaccharide region (Figure 3H).

Block scores of the multi-block models established using different spectral regions show that green snow bacteria grown in complex BHI medium and synthetic minimal media have various cellular chemical profiles probed by FTIR spectroscopy (Figure 4, Figure 5, Figure 6 and Figure 7). Thus, similarity of the lipid profile of *Rhodococcus*, *Leifsonia*, and *Pseudomonas* isolates grown on BHI agar was higher than when grown on synthetic minimal media (Figure 4 and Figure 5). According to the loading plots, such a variation in lipids when bacteria were grown on different synthetic minimal media is associated mainly with the shifts of the ester peak (C=O) between 1743 cm^−1^ and 1739 cm^−1^ depending on the media. Thus, for bacteria grown on BHI and XGU media, the ester peak was at 1743 cm^−1^, for MGU at 1741 cm^−1^ and for MGY and XGY at 1739 cm^−1^ (Figure 8A). In addition, loading plots of the lipid region show differences in intensity for the –C–H stretching bands of –CH_3_ and >CH_3_ and –CH_2_ in the region 3050–2800 cm^−1^ and for the C=O stretching in free fatty acids at 1710 cm^−1^ (Figure 8A).

When using mixed spectral region, we also observed a higher similarity of the biochemical profile for the cells grown on BHI and the cells grown on the minimal media were more distinctly different (Figure 5). Loading plots of this region show that lipid-associated bands such as –C–H stretching bands (1408 cm^−1^ and 1377 cm^−1^) and PO^−2^ asymmetric stretching of phosphodiesters in phospholipids at ~1230 cm^−1^ (PC1) and ~1250 cm^−1^ (PC2) are responsible for the distinctions of the cells grown on the minimal media (Figure 8B). Interestingly, cells cultivated on BHI showed different peaks identified as significant in the Amide III region (1350–1280 cm^−1^) (Figure 8B).

Alike lipids, the protein profile was significantly less different when these isolates were grown on BHI agar, while growth on some minimal media provided more protein-related differences as for example in the case of MGU and MGY (Figure 6). The loading plot for the protein region did not identify anything except difference in intensities and some shifts for Amide I (1620–1700 cm^−1^) and Amide II (~1550 cm^−1^) regions, indicating a change in the protein structure and profile (Figure 8C). Further, it is obvious that the lipid profile of *Arthrobacter*, *Pseudomonas*, and *Rhodococcus* isolates differs distinctly when grown on the synthetic minimal media (Figure 4A–D and Figure 5A–D), while less significant protein profile differences could be observed between *Arthrobacter*, *Pseudomonas*, and *Rhodoccocus* isolates grown on MGU and XGU media (Figure 6A,C).

The polysaccharide profile of *Arthrobacter* and *Rhodococcus* isolates grown on different media was very similar and only MGU and XGU media provided a slightly different biochemical profile for the cells (Figure 7A,C). Meanwhile *Pseudomonas* isolates showed to have a very distinct polysaccharide composition on all media. Loading plots of the polysaccharide region showed P=O stretching peak at 1078 cm^−1^ related to phospholipid and phosphodiester compounds [71] and some peaks of C–O–C and C–O ring vibrations in various polysaccharides (1120 cm^−1^, 1037 cm^−1^, and 980 cm^−1^) as the most significant (Figure 8D).

The cellular chemical profile of the green snow isolates of genus *Arthrobacter* grown on minimal agar was more strongly affected by different temperatures than when grown on complex BHI agar, while the differences between various minimal media were less significant. Growth at different temperatures showed a strain-specific effect on the chemical profile of the green snow bacteria where some isolates were affected to a higher extent and others to a lesser extent. Proteins showed to be quite consistent at different temperature and nutrient conditions, except for the XGU medium. *Rhodococcus* and *Pseudomonas* isolates showed quite consistent cell chemistry, which was not very affected by the temperatures, while all *Arthrobacter* isolates had a considerable change in the lipid and polysaccharide profile when grown on minimal agar at 25 °C and some isolates at 10 °C and 18 °C (Figure 4A–D and Figure 7A–D). *Rhodococcus* isolates and two overlapping isolates of *Leifsonia* and *Pseudomonas* had some temperature-induced changes in polysaccharides when grown at 4 °C (Figure 7) and in proteins when grown on XGU agar (Figure 6C). Lipids and polysaccharides of the isolate *Leifsonia antarctica* BIM B-1671 showed to be affected by the temperatures and nutrient alterations, while proteins were quite stable (Figure 4, Figure 5, Figure 6 and Figure 7).

### 3.3. Genus Specific Responses to Fluctuations in Temperature and Nutrient Conditions

**Figure 9:**
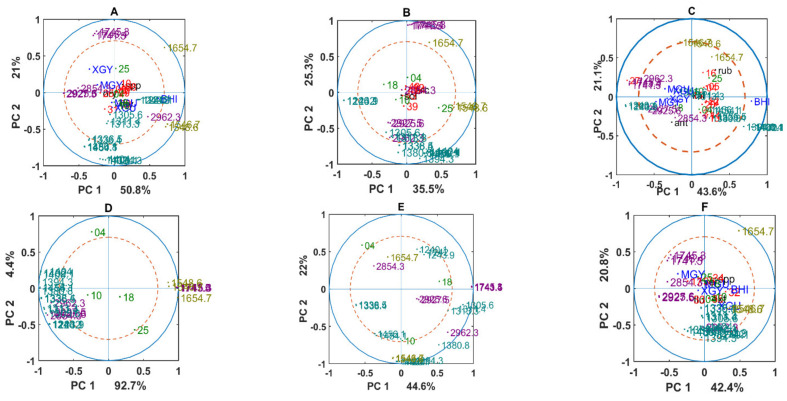

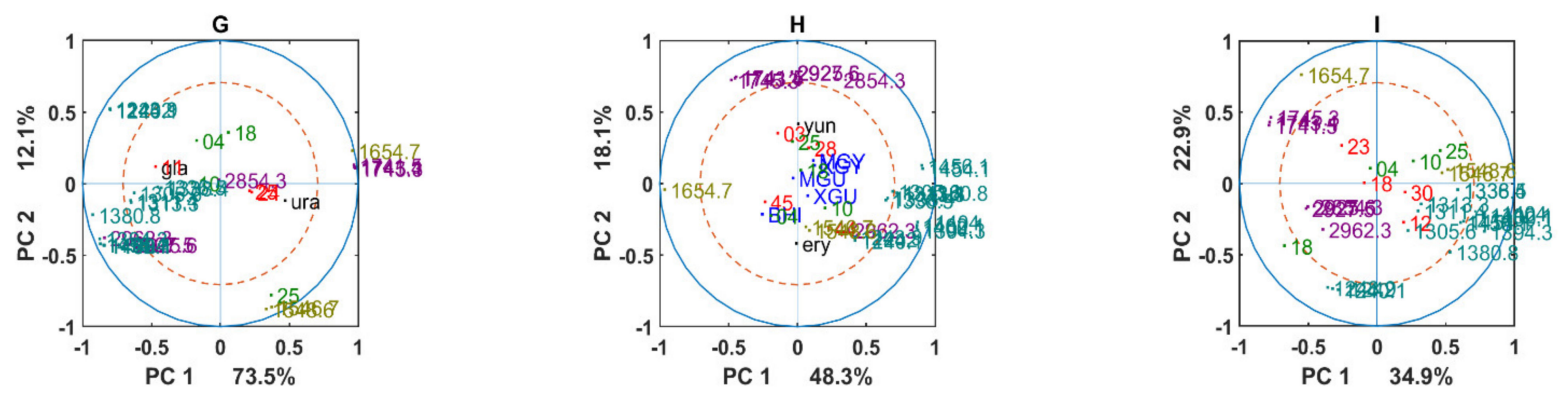
Correlation loading plots for PC1/PC2 for the genera: (**A**) *Arhtrobacter*; (**B**) *Cryobacterium*; (**C**) *Leifsonia*; (**D**) *Paeniglutamicibacter*; (**E**) *Polaromonas*; (**F**) *Pseudomonas*; (**G**) *Psychrobacter*; (**H**) *Rhodococcus*; (**I**) *Salinibacterium*. Red—isolate number; black—species; blue—media, green—temperature; purple—lipid/ester region; teal—mixed region; olive—protein region. Plots (**B**,**D**,**E**,**G**,**I**) contain no information about media since these genera were not growing on minimal media and the data are from bacteria grown on BHI agar medium.

To understand cellular phenotypic responses represented by the change in the chemical profile introduced by different temperature and nutrient conditions, we performed correlation analysis using standard PCA. Correlation analysis was done separately for each genus, pulling all the spectra of different media together. Correlation loading plots allow representation of the correlations among all variables available for the analysis, including spectral variables (peaks), design variables (media, temperature, bacterial genera, and strains) and any other variables available for the samples. We are especially interested to see the influence of temperature fluctuations for each genus and each isolate. When analyzing correlation loading plots presented in Figure 9, we evaluated correlation between nutrients/temperature conditions and selected IR peaks, which is an indication of change in the amount and profile of the corresponding chemical components. It is important to mention that genera *Cryobacterium*, *Paeniglutamicibacter*, *Polaromonas*, *Psychrobacter*, and *Salinibacterium* were only grown on BHI medium. Therefore, a parameter representing medium is missing on the correlation loading plots for these genera. The effect of temperature was observed for the genera which were grown on BHI only. Isolates from the genera *Arthrobacter*, *Pseudomonas*, *Rhodococcus*, and *Leifsonia,* which were able to grow on all media, are much more strongly affected by the media differences. Therefore, all temperatures are close to the middle of the plot. It can be seen that the cellular chemical profile of bacterial isolates was affected by temperature and nutrients differently, and some bacteria, as for example chemical profile of *Arthrobacter*, *Pseudomonas, Leifsonia*, and *Rhodococcus,* did not show any correlation between the temperatures they were exposed to (Figure 9A,C,F,H). Interestingly, these isolates did also not show some changes in the chemical cellular profile when grown on different nutrients (Figure 9A,F,H). Some negative correlation between BHI and XGY media associated with lipid and protein related bands indicating differences in the total lipid content and lipid profile in the cells (1741 cm^−1^, 1743 cm^−1^, 1745 cm^−1^, 2925 cm^−1^, 2927 cm^−1^) and in the protein profile (1546 cm^−1^ and 1548 cm^−1^) was observed for *Arthrobacter* (Figure 9A). For *Leifsonia* in Figure 9C, some negative correlation between BHI and MGY media associated with lipid and polysaccharide related bands indicating difference in total lipid content and lipid profile in the cells (1741 cm^−1^, 1743 cm^−1^, 1745 cm^−1^, 2925 cm^−1^, 2927 cm^−1^, 2962 cm^−1^) and polysaccharide profile (1394 cm^−1^, 1249 cm^−1^) was observed.

The most significant changes of the cellular chemical phenotype were observed when bacteria were exposed to very high and very low temperatures such as 25 °C and 4 °C. Thus, being exposed to 25 °C contributes to the possible changes in the protein and polysaccharide content and profile, as a clear correlation between these temperatures and protein related bands 1546 cm^−1^ and 1548 cm^−1^ and negative correlation with polysaccharide related bands 1240–1243 cm^−1^ was observed for *Cryobacterium*, *Paeniglutamicibacter*, and *Psychrobacter* (Figure 9B,D,G). The low growth temperature 4 °C often shows correlation with the wavenumber related to –CH_2_ and –CH_3_ stretching in lipids (2854 cm^−1^, 2962 cm^−1^, 2927 cm^−1^, 1394–1405 cm^−1^) as it was observed for *Cryobacterium*, *Polaromonas*, *Psychrobacter*, and *Salinibacterium* (Figure 9B,E,G,I). Interestingly, temperatures 10 °C and 18 °C had the strongest effect on *Polaromonas* and *Salinibacterium* isolates, which showed changes in lipid, polysaccharides, and protein composition (Figure 9E,I).

## 4. Discussion

Temperature and nutrients’ availability fluctuations are some of the main factors affecting the microbiome of polar regions. For understanding the influence of these parameters on the growth ability and cellular chemical profile of the Antarctic fast-growing green snow bacteria, we have selected two groups of cultivation media including rich complex and synthetic minimal media and four temperatures ranging from 4 to 25 °C. As a complex and rich medium, we used BHI agar, which is based on the brain-heart infusion and containing all macro- and micronutrients, and as synthetic minimal media we used a set of media containing two types of carbon source (glucose or glycerol), inorganic nitrogen, phosphorus, and some salts. These media and range of temperatures were chosen to mimic the availability of two nutrients and temperature scenarios in Antarctica, where minimal media and lower temperatures of 4 °C and 10 °C represent the current low nutrients’ availability and temperature scenario, and where rich complex medium and higher temperatures of 18 °C and 25 °C represent the scenario of elevated nutrients availability and temperature in the case of active snow melting due to the global temperature increase. In addition, these conditions are relevant for exploring the biotechnological potential of these bacteria.

The obtained results show that most of the Antarctic green snow bacteria grow on the rich complex medium, while only a limited number of the isolates were able to grow on the synthetic minimal media. In addition, bacteria grown on the synthetic minimal media were characterized by smaller colonies and longer growth time. This could be an indication of a high level of nutrients’ requirements for the studied green snow bacteria, which in the case of minimal media were obviously lacking, for example some vitamins, trace elements, amino acids, etc. [67,69,72,73]. Further, we observed that most of the bacteria grown on the minimal media were able to utilize both glucose and glycerol, indicating that these bacteria could possess some metabolic flexibility of a carbon source utilization, as previously reported [72,74,75,76].

The studied green snow bacteria could be considered as psychrotolerant as the best growth ability with the isolated colonies of a good size was observed at 18 °C. They could be characterized by a wide range of temperature tolerances; however, growth at lower and higher temperatures were suboptimal. It is important to note that growth reduction at lower temperatures (4 °C and 10 °C) was less significant than at a higher temperature (25 °C). It is well known that the size of colonies are determined by the individual cell metabolic kinetics, enzymatic activity, and cell population responses to environmental conditions [77]. This could be one of the main reasons of the observed low bacterial activity in green snow at lower temperatures. In a scenario such as global warming, with the moderate temperature increase and higher snow melting rate, we may expect elevated nutrient availability in green snow areas, subsequently causing an increase in bacterial activity, while high temperatures such as 25 °C will negatively impact the green snow bacterial populations by inhibiting their metabolic kinetics, which may result in the disappearance of some species.

The temperature-induced growth response of the studied green snow bacteria was genus and species-specific. Thus, among the studied green snow bacteria, almost all *Pseudomonas* isolates showed equally good growth ability on all tested media and temperatures. The isolate *Pseudomonas* sp. BIM B-1635 was characterized by a lower growth ability at 4 °C and 10 °C when cultivated on BHI agar and absence of growth when cultivated on minimal media. Therefore, we assume that this isolate is distinctly different from other *Pseudomonas* isolates. Such growth behavior is common for *Pseudomonas* bacteria as they are usually characterized by a wide growth temperature tolerance and are able to grow on complex and synthetic media [78]. Thus, it has been previously reported that the optimum growth temperature for most of the *Pseudomonas* species is 28 °C, while they can tolerate temperatures as low as 4 °C and as high as 45 °C [79]. Unusual growth ability was observed for *Arthrobacter* bacteria, which showed good growth at 4–18 °C and reduced growth at 25 °C, while temperatures of 20–30 °C were often reported as optimum for these bacteria isolated from non-polar regions [80,81]. The same was observed for *Psychrobacter* and *Salinibacterium* bacteria, for which the often reported optimum temperature is above 20 °C [68,82,83]. Interesting results were observed for *Leifsonia* isolates, where *Leifsonia antarctica* BIM B-1671 did not grow on minimal media while other isolates did. According to the previously reported studies, most of the *Leifsonia* species usually grow well on media containing rich organic nitrogen sources—yeast extract or peptone—but some species require the presence of growth factors such as amino acids or vitamins [84]. Thus, we can hypothesize that isolate *Leifsonia antarctica* BIM B-1671 belongs to another species.

High-throughput FTIR spectroscopy combined with the multivariate data analysis was utilized to investigate temperature and nutrients-induced changes of the cellular biochemical profile of the green snow bacteria. FTIR spectroscopy is a next-generation phenotypic technique widely used for rapid, non-invasive biochemical phenotyping of microorganisms [32,85,86]. In our previous study we utilized FTIR for total cellular biochemical phenotyping of the green snow bacteria grown on BHI agar [9]. In this study, we applied FTIR for profiling cellular chemical responses towards temperature and nutrients fluctuations. Multi-block analysis of IR spectra was used for understanding phenotypic relationships between the isolates grown on different temperatures and nutrients and identifying the most significant chemical components involved. In the multi-block analysis we used spectra of bacteria grown on different media to compare and combine the information.

The analysis showed that the chemical profile differences related to phylogeny were observed among bacteria grown on BHI medium. For example: (1) Gram-negative bacteria represented by genera *Psychrobacter*, *Pseudomonas*, and *Polaromonas* were clearly separated from the Gram-positive genera; (2) *Rhodococcus* isolates clustered out of the main cloud because they phylogenetically belong to the order *Mycrobacteriales*, while other genera belong to the order *Micrococcales*; (3) overlapping of genera *Leifsonia* and *Salinibacterium* can be explained by the previously reported phylogenetic similarity between these two genera, especially between *Salinibacterium* species and species *Leifsonia rubra* and *Leifsonia aurea* [69], and (4) the overlapping of *Paeniglutamicibacter* and *Arthrobacter* isolates could be due to the re-classification of *Arthrobacter antarcticus* to *Paeniglutamicibacter antarcticus* [87]. The clearest clustering according to the phylogeny was observed using lipid and mixed regions, where the fatty acid chain length, total content, and profile of lipids are the most significant parameters according to the loading plots. The differences observed in lipid IR region is in accordance with the previously reported findings, where it has been shown that membrane fatty acids could be considered as a phenotypic biomarkers of the bacterial polyphasic taxonomy, which integrates phylogenetic relationships with phenotypic marker analysis [88]. Interestingly, the FTIR protein profile showed much smaller contributions to the phylogenetic distribution of the studied bacteria. This could be due to protein profile probed by FTIR being much less specific to be used as a taxonomic biomarker. The protein profile used for polyphasic taxonomy is usually measured by gel electrophoresis techniques [89]. Multi-block score plots showed that isolate *Pseudomonas fluorescence* BIM B-1668 clustered out from all other *Pseudomonas* isolates and overlapped with isolate *Leifsonia antarctica* BIM B-1671 could be explained by potentially sequencing-related misidentification. Taxonomic differences in polysaccharides are more known for fungi, and in the case of bacteria, differences in the polysaccharides were reported mainly associated with the cell-wall structure [66]. In this study, we show that the phylogenetic clustering of the green snow bacteria is little associated with changes in polysaccharides.

Under different nutrients and temperature conditions, the most considerable changes in the cellular chemical profile were associated with different media and, when only one medium was used, then low and high temperatures had a strong effect, which was often associated with lipid-related FTIR bands and lesser associated with protein and polysaccharide bands. The correlation analysis indicated that the whole chemical composition is changing under different nutrients and temperature growth conditions. Correlation analysis showed that nutrients are clearly a stronger parameter influencing chemical changes in the cells, followed by temperature. It is known that temperature may considerably affect the amount and profile of the intracellular metabolites in microorganisms [90,91,92]. Psychrophilic bacteria have a series of adaptation mechanisms for surviving in cold environments, and one of them is related to the alteration in the membrane fatty acids’ content and composition [93] and the expression of heat/cold shock proteins [94,95]. For example, it was reported that Gram-negative bacteria grown at low temperatures (5 °C and 15 °C) have a high quantity of n-MUFAs, which decreases when grown at high temperatures (25 °C and 35 °C), while for Gram-positive bacteria a high level of br-SFAs in the cell membrane was recorded at high temperatures (25 °C and 35 °C), which decreased at lower temperatures [19]. In addition, it is worth mentioning that changes in lipids were generally observed for all green snow isolates, where the main correlation was with bands of ester bond stretching bands 1741 cm^−1^, 1743 cm^−1^, and 1745 cm^−1^, indicating a possible change in the total lipid content and, possibly, a type of produced lipids. In addition, it was correlated/negatively correlated with CH_2_ and CH_3_ stretching bands, indicating a change in the lipids’ length and branching. Thus, it could be concluded that lipids might be one of the main cellular building blocks involved in the nutrients/temperature adaptations. Further, the response to temperature fluctuations was more pronounced when bacteria were grown on the synthetic minimal media than on complex BHI agar. This is in accordance with the previously published reports, which showed that cultivation in rich complex media alters mainly the total lipid content and the length of the fatty acids chain, but there are no changes in a proportion of branched-chain fatty acids and fatty acid profile in general. In minimal synthetic media, though, there are changes in the pattern of fatty acids branching regarding the amount of branched-chain fatty acids compared to straited-chain saturated and unsaturated fatty acids [96]. However, in general, temperature fluctuations affected cellular chemical composition to a lower extent than nutrients.

Our study shows that under the temperature rising conditions, green snow bacteria will contribute to the microbial flora of Antarctic regions as they are able to grow at higher temperatures and can utilize different carbon sources. Due to the fact that the overall cellular chemical profile was changing to a different extent under various temperatures, further study would be needed to understand in detail how these green snow bacteria alter their chemical composition. Changes in lipid profile and content are especially interesting.

## 5. Conclusions

In conclusion, we identified that Antarctic green snow bacteria are psychrotolerant and able to grow at lower and higher temperatures. All bacteria were able to grow on complex rich medium, and only few of them grew on minimal synthetic media. Alteration in nutrients induced bigger changes in the cellular chemical profile than temperature. The most significant taxonomic differences were observed in lipids, while smaller differences were observed in proteins and polysaccharides. It could be concluded that lipids might be one of the main cellular building blocks involved in the nutrients/temperature adaptations.

## Figures and Tables

**Table 1:** List of Antarctic bacterial isolates used in the study.

Bacterial Isolate Name	Collection No/Samples Number [9]
*Arthrobacter cryoconiti*	BIM B-1627/G.S.9
*Arthrobacter oryzae*	BIM B-1663/G.S.26
*Arthrobacter* sp.	BIM B-1624/G.S.6
*Arthrobacter* sp.	BIM B-1625/G.S.7
*Arthrobacter* sp.	BIM B-1626/G.S.8
*Arthrobacter* sp.	BIM B-1628/G.S.10
*Arthrobacter* sp.	BIM B-1664/G.S.29
*Arthrobacter* sp.	BIM B-1666/G.S.31
*Arthrobacter* sp.	BIM B-1656/G.S.37
*Cryobacterium arcticum*	BIM B-1619/G.S.1
*Cryobacterium arcticum*	BIM B-1620/G.S.2
*Cryobacterium soli*	BIM B-1658/G.S.39
*Cryobacterium soli*	BIM B-1659/G.S.40
*Cryobacterium soli*	BIM B-1677/G.S.41
*Cryobacterium soli*	BIM B-1675/G.S.43
*Leifsonia antarctica*	BIM B-1631/G.S.13
*Leifsonia antarctica*	BIM B-1632/G.S.14
*Leifsonia antarctica*	BIM B-1637/G.S.19
*Leifsonia antarctica*	BIM B-1638/G.S.20
*Leifsonia antarctica*	BIM B-1639/G.S.21
*Leifsonia antarctica*	BIM B-1669/G.S.22
*Leifsonia antarctica*	BIM B-1671/G.S.27
*Leifsonia kafniensis*	BIM B-1633/G.S.15
*Leifsonia rubra*	BIM B-1622/G.S.4
*Leifsonia rubra*	BIM B-1623/G.S.5
*Leifsonia rubra*	BIM B-1634/G.S.16
*Paeniglutamicibacter antarcticus*	BIM B-1657/G.S.38
*Polaromonas* sp.	BIM B-1676/G.S.42
*Pseudomonas extremaustralis*	BIM B-1672/G.S.35
*Pseudomonas fluorescens*	BIM B-1668/G.S.33
*Pseudomonas* sp.	BIM B-1635/G.S.17
*Pseudomonas* sp.	BIM B-1667/G.S.32
*Pseudomonas* sp.	BIM B-1673/G.S.34
*Pseudomonas versuta*	BIM B-1674/G.S.36
*Psychrobacter glacinicola*	BIM B-1629/G.S.11
*Psychrobacter urativorans*	BIM B-1655/G.S.24
*Psychrobacter urativorans*	BIM B-1662/G.S.25
*Rhodococcus yunnanensis*	BIM B-1621/G.S.3
*Rhodococcus yunnanensis*	BIM B-1670/G.S.28
*Rhodococcus erythropolis*	BIM B-1660/G.S.44
*Rhodococcus erythropolis*	BIM B-1661/G.S.45
*Salinibacterium* sp.	BIM B-1630/G.S.12
*Salinibacterium* sp.	BIM B-1636/G.S.18
*Salinibacterium* sp.	BIM B-1654/G.S.23
*Salinibacterium* sp.	BIM B-1665/G.S.30

**Table 2:** Chemical composition of the cultivation media used in the study.

Composition of Salt Solution (g/L)	MGU	MGY	XGU	XGY	BHI
Na_2_HPO_4_	24	24	-	-	-
KH_2_PO_4_	12	12	4	4	-
K_2_HPO_4_	-	-	12	12	-
NaCl	2.5	2.5	-	-	-
NH_4_Cl	5	5	20	20	-
NH_4_NO_3_	-	-	4	4	-
Na_2_SO_4_ × 10H_2_O	-	-	8	8	-
MgSO_4_ × 7H_2_O	-	-	0.4	0.4	-
Commercial powder	-	-	-	-	37
Media composition (mL)					
Salt solution	100	100	100	100	-
20% agar	300	300	300	300	1000
0.1 M CaCl_2_	4	4	-	-	-
0.1 M MgSO_4_	4	4	-	-	-
20% glucose	4	-	4	-	-
20% glycerol	-	4	-	4	-

**Table 3:** Growth ability of the green snow bacteria on different media and temperatures (samples marked with colors based on grade scale: 0–2—red, 3–4—orange, 5–6—yellow, 7–8—blue, 9–10—green).

		*Arthrobacter*										
		1624 * G.S.6	1625 G.S.7	1626 G.S.8	1627 G.S.9	1628 G.S.10	1663 G.S.26	1664 G.S.29	1666 G.S.31	1656 G.S.37		
BHI	4 °C											
	10 °C											
	18 °C											
	25 °C											
XGU	4 °C											
	10 °C											
	18 °C											
	25 °C											
XGY	4 °C											
	10 °C											
	18 °C											
	25 °C											
MGU	4 °C											
	10 °C											
	18 °C											
	25 °C											
MGY	4 °C											
	10 °C											
	18 °C											
	25 °C											
		*Pseudomonas*							*Rhodococcus*			
		1635 G.S.17	1667 G.S.32	1668 G.S.33	1673 G.S.34	1672 G.S.35	1674 G.S.36		1621 G.S.3	1670 G.S.28	1660 G.S.44	1661 G.S.45
BHI	4 °C											
	10 °C											
	18 °C											
	25 °C											
XGU	4 °C											
	10 °C											
	18 °C											
	25 °C											
XGY	4 °C											
	10 °C											
	18 °C											
	25 °C											
MGU	4 °C											
	10 °C											
	18 °C											
	25 °C											
MGY	4 °C											
	10 °C											
	18 °C											
	25 °C											
		*Cryobacterium*							*Salinibacterium*			
		1619 G.S.1	1620 G.S.2	1658 G.S.39	1659 G.S.40	1677 G.S.41	1675 G.S.43		1630 G.S.12	1636 G.S.18	1654 G.S.23	1665 G.S.30
BHI	4 °C											
	10 °C											
	18 °C											
	25 °C											
XGU XGY MGU MGY	4 °C											
	10 °C											
	18 °C											
	25 °C											
		*Leifsonia*										
		1622 G.S.4	1623 G.S.5	1631 G.S.13	1632 G.S.14	1633 G.S.15	1634 G.S.16	1637 G.S.19	1638 G.S.20	1639 G.S.21	1669 G.S.22	1671 G.S.27
BHI	4 °C											
	10 °C											
	18 °C											
	25 °C											
XGU XGY MGU MGY	4 °C											
	10 °C											
	18 °C											
	25 °C											
		*Psychrobacter*				*Paeniglutamicibcter antarcticus* 1657 G.S.38				*Polaromonas* sp. 1676 G.S.42		
		1629 G.S.11	1655 G.S.24	1662 G.S.25								
BHI	4 °C											
	10 °C											
	18 °C											
	25 °C											
XGU XGY MGU MGY	4 °C											
	10 °C											
	18 °C											
	25 °C											

* Numbers are according to the registration number BIM B-XXXX at the Belarussian Collection of Non-pathogenic Microorganisms (Institute of Microbiology of the National Academy of Sciences of Belarus) (Table 1).

## Data Availability

Data provided in this paper are available in the Zenodo repository at https://doi.org/10.5281/zenodo.6444884 (accessed on 9 March 2022).

## Supplementary Materials

The following supporting information can be downloaded at: https://www.mdpi.com/article/10.3390/biology11060890/s1, Table S1: Cultivation time of green snow bacteria at different temperature and nutrient conditions (grey color indicates the absence or very little growth with a growth ability score lower than 4).
