# Supplementary material for: Temperature- and Nutrients-Induced Phenotypic Changes of Antarctic Green Snow Bacteria Probed by High-Throughput FTIR Spectroscopy
Source: Biology (Basel). 2022 Jun 9;11(6):890. doi: 10.3390/biology11060890 (PMC9220083; doi:10.3390/biology11060890)
Supplement: Supplementary file 1 [file biology-11-00890-s001.zip › biology-1658562-supplementary.pdf]

Table S1. Cultivation time of green snow bacteria at different temperature and nutrient conditions (gray color indicates the absence or very little growth with the growth ability score lower than 4).

Sample	Cultivation time in days																			
	4°C					10°C					18°C					25°C				
	BHI	MGU	MGY	XGU	XGY	BHI	MGU	MGY	XGU	XGY	BHI	MGU	MGY	XGU	XGY	BHI	MGU	MGY	XGU	XGY
Arthrobacter																				
BIM B-1627	7	12	12	12	12	4	7	7	7	7	4	8	8	8	8	7				
BIM B-1663	7					4	7	7	7	7	4	8	8	8	8	7	9	9	9	9
BIM B-1624	7	12	12	12	12	4	7	7	7	7	4	8	8	8	8	7	9	9	9	9
BIM B-1625	7	12	12	12	12	4	7	7	7	7	4	8	8	8	8	7	9	9	9	9
BIM B-1626	7	12	12	12	12	4	7	7	7	7	4	8	8	8	8	7	9	9	9	9
BIM B-1628	7	12	12	12	12	4	7	7	7	7	4	8	8	8	8	7	9	9	9	9
BIM B-1664	7	12	12	12	12	4	7	7	7	7	4	8	8	8	8	7	9	9	9	9
BIM B-1666	7	12	12	12	12	4	7	7	7	7	4	8	8	8	8	7				
BIM B-1656	7	12	12	12	12	4	7	7	7	7	4	8	8	8	8	7	9	9	9	9
Cryobacterium																				
BIM B-1619	10					7					4					7				
BIM B-1620	10					7					4					7				
BIM B-1658	10					7					4					7				
BIM B-1659	10					7					4					7				
BIM B-1677	10					7					4					7				
BIM B-1675	10					7					4					7				
Leifsonia																				
BIM B-1631	11					9					5					9				
BIM B-1632	11					9					5					9				
BIM B-1637	11					9					5					9				
BIM B-1638	11					9					5					9				
BIM B-1639	11					9					5					9				
BIM B-1669	11					9					5					9				
BIM B-1671	11	11	11	11	11	9	9	9	9	9	5	10	10	10	10	9				
BIM B-1633	11					9					5					9				
BIM B-1622	11					9					5					9				
BIM B-1623	11					9					5					9				
BIM B-1634						9					5					9				
Salinibacterium																				
BIM B-1630											5					9				
BIM B-1636						9					5					9				
BIM B-1654	11					9					5					9				
BIM B-1665						9					5					9				

Rhodococcus																				
BIM B-1621	11					9					3					5				
BIM B-1670	11	12	12	12	12	9	10	10	10	10	3	8	8	8	8	5	9	9	9	9
BIM B-1660	7	12	12	12	12	5	6	6	6	6	3	7	7	7	7	3	5	5	5	5
BIM B-1661	7	12	12	12	12	5	6	6	6	6	3	7	7	7	7	3	5	5	5	5
Paeniglutamicibacter																				
BIM B-1657	10					7					4					6				
Polaromonas																				
BIM B-1676	11					10					10									
Pseudomonas																				
BIM B-1668	7	9	9	9	9	4	6	6	6	6	3	4	4	4	4	3	3	3	3	3
BIM B-1672	7	8	8	8	8	4	5	5	5	5	3	4	4	4	4	3	3	3	3	3
BIM B-1674	7	8	8	8	8	4	5	5	5	5	3	4	4	4	4	3	3	3	3	3
BIM B-1667	7	9	9	9	9	4	6	6	6	6	3	4	4	4	4	3	3	3	3	3
BIM B-1635	10					4					3	4	4	4	4	3	3	3	3	3
BIM B-1673	7	8	8	8	8	4	5	5	5	5	3	4	4	4	4	3	3	3	3	3
Psychrobacter																				
BIM B-1629	11					9					3									
BIM B-1655	11					9					3					9				
BIM B-1662	11					9					3					9				
